# Detection of tissue coagulation by decorrelation of ultrasonic echo signals in cavitation-enhanced high-intensity focused ultrasound treatment

**DOI:** 10.1186/s40349-016-0060-0

**Published:** 2016-04-14

**Authors:** Shin Yoshizawa, Keiko Matsuura, Ryo Takagi, Mariko Yamamoto, Shin-ichiro Umemura

**Affiliations:** Graduate School of Engineering, Tohoku University, Sendai, 980-8579 Japan; Graduate School of Biomedical Engineering, Tohoku University, Sendai, 980-8579 Japan

**Keywords:** HIFU, Cavitation, Ultrasound imaging, Coagulation detection, Decorrelation

## Abstract

**Background:**

A noninvasive technique to monitor thermal lesion formation is necessary to ensure the accuracy and safety of high-intensity focused ultrasound (HIFU) treatment. The purpose of this study is to ultrasonically detect the tissue change due to thermal coagulation in the HIFU treatment enhanced by cavitation microbubbles.

**Methods:**

An ultrasound imaging probe transmitted plane waves at a center frequency of 4.5 MHz. Ultrasonic radio-frequency (RF) echo signals during HIFU exposure at a frequency of 1.2 MHz were acquired. Cross-correlation coefficients were calculated between in-phase and quadrature (IQ) data of two B-mode images with an interval time of 50 and 500 ms for the estimation of the region of cavitation and coagulation, respectively. Pathological examination of the coagulated tissue was also performed to compare with the corresponding ultrasonically detected coagulation region.

**Results:**

The distribution of minimum hold cross-correlation coefficient between two sets of IQ data with 50-ms intervals was compared with a pulse inversion (PI) image. The regions with low cross-correlation coefficients approximately corresponded to those with high brightness in the PI image. The regions with low cross-correlation coefficients in 500-ms intervals showed a good agreement with those with significant change in histology.

**Conclusions:**

The results show that the regions of coagulation and cavitation could be ultrasonically detected as those with low cross-correlation coefficients between RF frames with certain intervals. This method will contribute to improve the safety and accuracy of the HIFU treatment enhanced by cavitation microbubbles.

## Background

High-intensity focused ultrasound (HIFU) is a noninvasive technique for thermal ablation of solid tumors and has already been used to treat fibroids and prostatic tumors. Ultrasound can be focused to a target tumor such as cancer to be thermally coagulated selectively [1]. However, unlike in an open surgery, the operator or surgeon cannot look at the tissue being or to be treated by a naked eye in real time. Therefore, a noninvasive technique to monitor thermal lesion formation is necessary to ensure the accuracy and safety of HIFU treatment.

Magnetic resonance imaging (MRI) and ultrasonic imaging are currently used for monitoring HIFU treatment and its therapeutic effects. MRI has the advantage in tissue temperature monitoring [2], but it lacks real-time monitoring capability especially for cavitation. Ultrasonic imaging is chosen in this study because of its higher spatial and temporal resolution at a much lower cost. A hyper-echoic change in a normal B-mode image at the focal point of HIFU has been used for estimating the coagulation area, but the change is faint unless bubbles are generated due to cavitation or boiling [3]. Slight changes in ultrasonic backscatter were observed corresponding to thermal coagulation as well as changes in the speed of sound and attenuation coefficient in a liver tissue [4]. In hepatic tissue, the relation between the change in ultrasonic backscatter and the slight increase in the hepatic cell concentration was shown, which was observed in the histological structure [5]. Taking this slight change into account, the method to estimate the coagulation by comparing two radio-frequency (RF) frames which correspond to the two B-mode images during HIFU exposure using the distribution of cross-correlation coefficient has been studied, and the decorrelation corresponding to coagulation was observed in the focal area of HIFU exposure even in cases that bubbles due to boiling or cavitation were not observed [6, 7]. The echo decorrelation imaging has been investigated also for the ultrasound monitoring of RF ablation [8].

With a typical equipment for current HIFU treatment, an ultrasound exposure at 4 MHz for 3 s can treat a volume of 2 × 2 × 10 to 3 × 3 × 12 mm^3^ [9], resulting in that the median operating time of 142 min (ranging 35–390 min), including cooling time between consecutive HIFU exposures, is needed to treat the median prostate volume of 21.9 cm^3^ (ranging 4.6–68.8 cm^3^) [10]. This long treatment time is thought to be a significant drawback of HIFU treatment. The enhancement of ultrasonic heating by cavitation bubbles is receiving a lot of attention to solve this problem [11–16]. Acoustic cavitation is the phenomenon in which microbubbles in the order of 0.1–10 μm in diameter are generated by acoustic irradiation. In HIFU treatment, cavitation bubbles can be generated and vibrated by ultrasound, can enhance the heating effect, and make the treatment time much shorter [17–21].

The objective of this study is to develop a noninvasive technique to monitor the thermal lesion formation, even in the existence of such cavitation bubbles. In this study, high-speed imaging by parallel beamforming is performed using ultrasound RF signals acquired during HIFU exposure, and the cross-correlation coefficient between the obtained RF frames is calculated according to the previous study [7]. Then, both coagulation and cavitation regions are detected as the regions of low correlation. They should be distinguished by changing the interval between the RF frames for cross-correlation because the order of the time constant is quite different for cavitation bubbles and coagulation.

## Methods

### HIFU exposure and RF data acquisition sequence

**Fig. 1:**
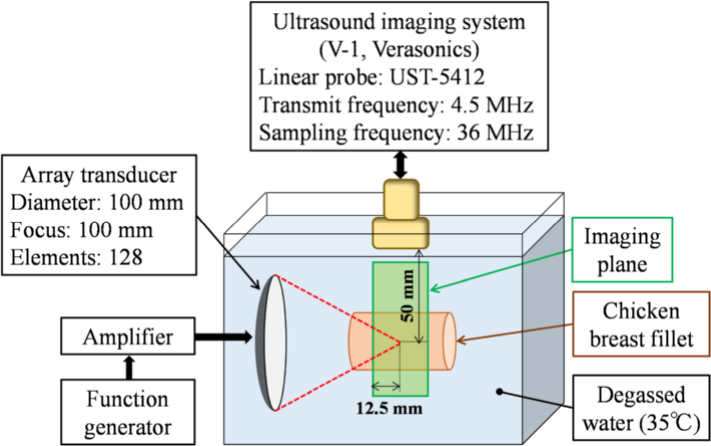
Schematic of experimental setup. HIFU was focused onto the chicken breast fillet in degassed water. The water temperature was kept at approximately 35 °C. A linear ultrasound probe was used to acquire RF data for B-mode images during HIFU exposure. The HIFU focal point was set at 50.0 mm from the probe surface in the depth direction and 12.5 mm from the left edge of the B-mode images in the lateral direction

Figure 1 shows a schematic of the experimental setup. The chicken breast fillet was excised and degassed in saline for 4 h to decrease remaining gaseous content in the tissue. It was because such contents can significantly reduce the cavitation threshold of the tissue. The tissue was restrained at 4 points each at the top and bottom. The water was kept at approximately 35 °C. HIFU was generated from the 128-channel array transducer (Imasonic, Voray sur l’Ognon, France) with both focal length and aperture diameter of 100 mm at a driving frequency of 1.2 MHz. The accurate position of its focal point was found by a needle hydrophone (HPM02, Precision Acoustics, Dorset, UK).

**Fig. 2:**
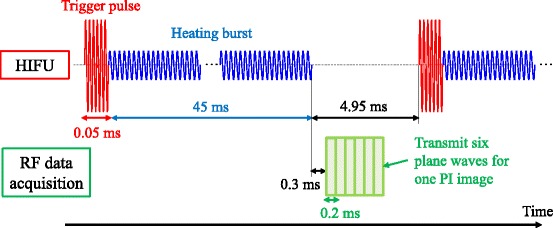
Sequence of HIFU exposure and RF data acquisition. A high-intensity short pulse, named as “trigger pulse,” for the generation of cavitation bubbles was immediately followed by a lower intensity longer duration burst, named as “heating burst,” for the bubble-enhanced heating. The intensity of heating burst is at a similar level as conventional HIFU. The sequence was repeated for 10 s. RF data were acquired during each HIFU intermission period of 4.95 ms

The sequence of HIFU exposure and RF data acquisition is shown in Fig. 2. The HIFU sequence, called “Triggered HIFU” [17–21], consisted of two kinds of exposure as follows. A high-intensity short pulse, named as “trigger pulse,” generates and grows cavitation bubbles first. Then, a lower intensity longer duration burst, named as “heating burst,” vibrates the cavitation bubbles to enhance the heating effect. The intensity of heating burst is at a similar level as conventional HIFU. As the result, a large volume of tissue can be efficiently coagulated [19–21]. In this study, a trigger pulse at a spatial-peak pulse-average intensity of 30 kW/cm^2^ with a duration of 0.05 ms was irradiated. It was immediately followed by a heating burst at a spatial-peak pulse-average intensity of 2 kW/cm^2^ with a duration of 45 ms. The intermission between consecutive HIFU exposures was 4.95 ms. The sequence was repeated 200 times, resulting in the total duration of the sequence of 10 s. RF signals were acquired during each HIFU intermission period.

### Ultrasound imaging

In this study, RF signals were obtained by combining two imaging ultrasound irradiation methods. First, plane wave transmission followed by parallel beamforming for high-speed ultrasonic imaging, at a frame rate higher than 5000 fps [22], was applied. To improve the contrast ratio of the B-mode image, multiple steered plane waves were transmitted, and the obtained ultrasonic images were coherently compounded [23, 24]. To visualize cavitation bubbles, “pulse inversion (PI)” [25] sequence was employed. Plane wave transmission was performed six times in total, with a separation angle of 6°, at −6°, 0°, and +6°. At each angle, twice transmission of one-cycle sine wave with opposite phases was performed. The phases of the first and second sine waves were 0° and 180°, respectively. The pulse repetition period of the six plane waves was 0.2 ms. The transmission center frequency and the sampling frequency were set at 4.5 and 36 MHz, respectively. These parameters may not be thoroughly optimized, so further optimization will be possible if necessary. The ultrasonic imaging plane was set so that it contained the axis of the HIFU beam as seen in Fig. 1. The HIFU focal point was 50.0 mm from the probe surface in the depth direction and 12.5 mm from the left edge of the B-mode images in the lateral direction. The peak-to-peak pressure of the transmitted plane waves was 2.0 MPa with a steering angle of 0° at a depth of 50.0 mm.

A programmable ultrasound imaging system (V-1 System, Verasonics, WA, USA) with a linear array probe (UST-5412, Hitachi Aloka Medical, Tokyo, Japan) was used for ultrasonic monitoring. The data of 200 RF frames were acquired at a frame rate of 20 fps, resulting in a total duration of 10 s. To avoid the echoes of HIFU, the first plane wave transmission was performed 0.3 ms after the heating waves stopped as seen in Fig. 2.

### Cross-correlation coefficient distribution

To estimate both coagulation and cavitation regions, a cross-correlation coefficient using block matching algorithm was calculated between image blocks in each of the two sets of in-phase and quadrature (IQ) data. A set of IQ data was calculated by parallel beamforming from three RF data which were acquired just after the three plane wave transmissions with angles of −6°, 0°, and +6° and a phase of 0°. The pixel size of the IQ data was 0.084 and 0.169 mm in depth and width, respectively. A block matching method was used to simultaneously compensate the tissue motion. The correlation coefficient was calculated as

1 $$ \begin{array}{c}\left|R\left(k,l\right)\right|=\frac{\left|{\displaystyle \sum_{i=1}^{M_i}}{\displaystyle \sum_{j=1}^{N_j}T*\left(i,j\right)I\left(i+k,j+l\right)}\right|}{\sqrt{{\displaystyle \sum_{i=1}^{M_i}}{\displaystyle \sum_{j=1}^{N_j}{\left|T\left(i,j\right)\right|}^2}}\sqrt{{\displaystyle \sum_{i=1}^{M_i}}{\displaystyle \sum_{j=1}^{N_j}{\left|I\left(i+k,j+l\right)\right|}^2}}}\\ {}\left(-{M}_k\le k\le {M}_k,\kern0.75em -{N}_l\le l\le\;{N}_l\right)\end{array} $$

First, a correlation window *T*(*i*,*j*) was chosen as a reference block in the reference frame, and then the block *I*(*i*,*j*) best matching the reference block was searched in a search region in the target frame. The sizes of the correlation window and search region were set to 0.76 by 0.84 mm (9 by 5 pixels) and 1.44 by 1.52 mm (17 by 9 pixels) respectively, that is, *M*_*i*_ = 9, *N*_*j*_ = 5, *M*_*k*_ = 8, and *N*_*l*_ = 4 in Eq. (1). The window producing the highest correlation coefficient with the reference window was searched within the search region.

2 $$ {R}_{\max }= \max \left[\left|R\left(k,l\right)\right|\right] $$

The maximum value of the correlation coefficient described by Eq. (2) was plotted as a function of the reference window position. Then, cross-correlation coefficients were calculated for the next reference window, which 50 % overlapped with the previous one for a sufficient spatial resolution. A motion-compensated distribution of correlation coefficient between the two entire images can thus be depicted. Both coagulation and cavitation regions were estimated by changing the interval between the RF frames for cross-correlation because the orders of magnitude of time constants of cavitation bubbles and tissue coagulation are quite different. In this study, the intervals to detect cavitation and coagulation were set to 50 and 500 ms, respectively.

### Pathologic examination

After a series of HIFU exposure and RF signal acquisition, samples were observed pathologically. They were frozen by use of liquid nitrogen within 5 h and cut in 10-μm-thick slices in the direction parallel to the imaging plane. A slice was obtained every 100 μm perpendicular to the plane, and the slice with the largest coagulation region was chosen and stained with hematoxylin and eosin (H&E). The muscle fiber atrophy due to thermal coagulation was observed [26], so the coagulation region was determined as a coarse region of atrophic muscle fibers in a low-power microscopic field.

### Estimation of the cross-correlation coefficient threshold

**Fig. 3:**
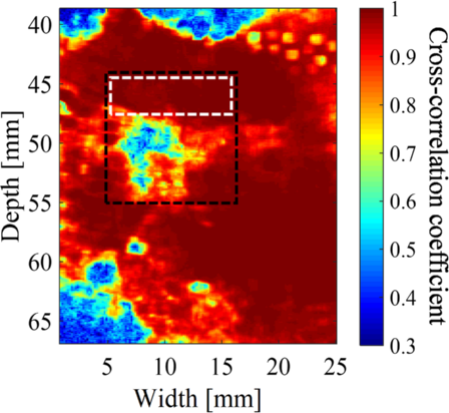
An example of cross-correlation coefficient map after HIFU exposure. The cross-correlation coefficients were calculated between two sets of IQ data using a block matching algorithm. The cross-correlation coefficients were slightly and significantly decreased in *white* and *black frame*, respectively

**Fig. 4:**
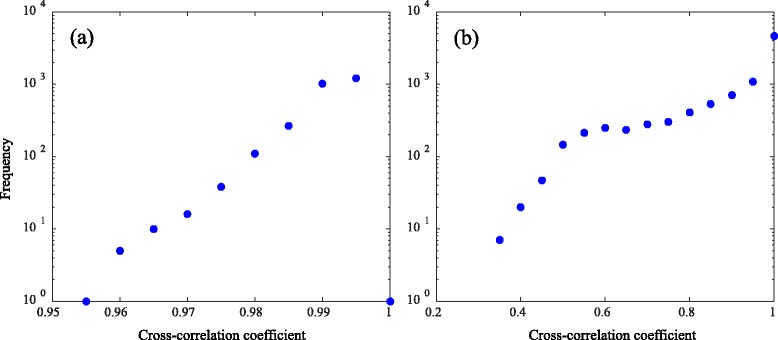
Histograms in white (**a**) and black (**b**) frame in Fig. 3. While the frequency almost monotonically increases in **a**, a local maximum at a cross-correlation coefficient of 0.6 is seen in **b**

Cross-correlation coefficients can be lowered by the temperature distribution in the tissue, the tissue deformation, and noises as well as the cavitation and coagulation phenomena. To estimate the regions of cavitation and coagulation from the distribution of the cross-correlation coefficient, it is important to set the coefficient threshold so that the influences of other phenomena are suppressed. In this study, it was determined from the regularity of the histogram of the number of pixels plotted against the cross-correlation coefficient. Figure 3 shows an example of the results of the cross-correlation coefficient distribution. The position of 0 mm in depth and 12.8 mm in width corresponds to the surface and center of the imaging probe, respectively. Histograms of the coefficients in the white and black frame in Fig. 3 are plotted in Fig. 4a, b, respectively. Two peaks at cross-correlation coefficients of 0.6 and 1.0 are seen in Fig. 4b. The two distributions around 0.6 and 1.0 in Fig. 4b presumably correspond to different phenomena, while only one in Fig. 4a. The distribution with a higher correlation coefficient than the other in Fig. 4b corresponds to the same phenomenon as the single peak in Fig. 4a, which should be different from either cavitation or tissue coagulation. The distribution with a lower correlation coefficient in Fig. 4b, on the other hand, presumably corresponds to such a phenomenon of interest, in this particular case, tissue coagulation. Therefore, by setting the cross-correlation coefficient threshold at the local minimum between the two peaks, the region of the phenomenon of interest should be successfully estimated with suppressing the influence of the phenomenon corresponding to the distribution with the higher correlation coefficient. The cross-correlation coefficient at the local minimum was 0.65 in this particular case.

## Results

### B-mode images

**Fig. 5:**
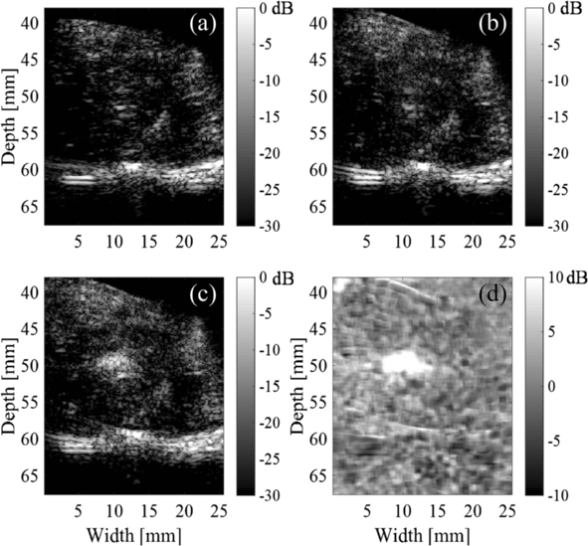
B-mode images of the tissue **a** before the start, **b** immediately after the start, and **c** end of HIFU exposure and **d** differential B-mode image between **c** and **b**. The propagation direction of HIFU is from left to right. The HIFU focal point was 50.0 mm in depth and 12.5 mm in width, which was measured with the needle hydrophone in water

Five tissue samples were used in this study. Figure 5a, b, c shows B-mode images of one of the tissues captured immediately before and after the start and end of a HIFU exposure. Figure 5c includes the area of tissue change. The propagation direction of HIFU is from left to right. The HIFU focal point was 50.0 mm in depth and 12.5 mm in width. A differential B-mode image between Fig. 5c and a is shown in Fig. 5d. The difference was obtained by averaging the power of the IQ data in 9 by 5 pixels and subtracting the logarithms of the two frames. In the differential B-mode image, an area with increased echo strength is seen around the HIFU focal spot. The area may contain both areas of tissue change and cavitation, which are difficult to distinguish from each other.

### Histology

**Fig. 6:**
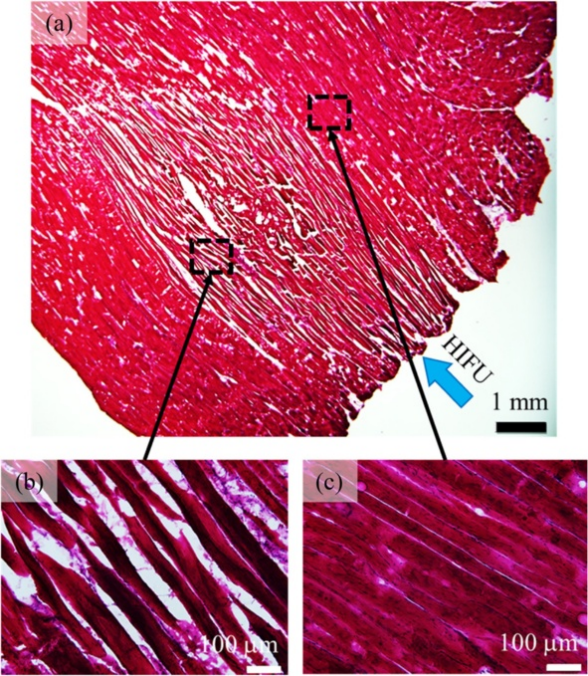
Slice of a tissue sample stained with H&E after HIFU exposure. **a** Whole coagulation region, **b** coagulation, and **c** normal regions magnified by 20 times

Figure 6a shows the sample of tissue stained with H&E. The tissue sample is the same that was used in Fig. 5. The coagulated and normal areas are magnified 20 times in Fig. 6b, c, respectively, where many muscle fibers are seen in each area. In Fig. 6b, each muscle fiber is narrower than those in Fig. 6c, and even cracks between fibers are seen as white stripes. This whitish area is regarded as the actual coagulation region. In this particular case, it has an area of about 26.0 mm^2^.

### Distribution of cross-correlation coefficient with 50-ms intervals

**Fig. 7:**
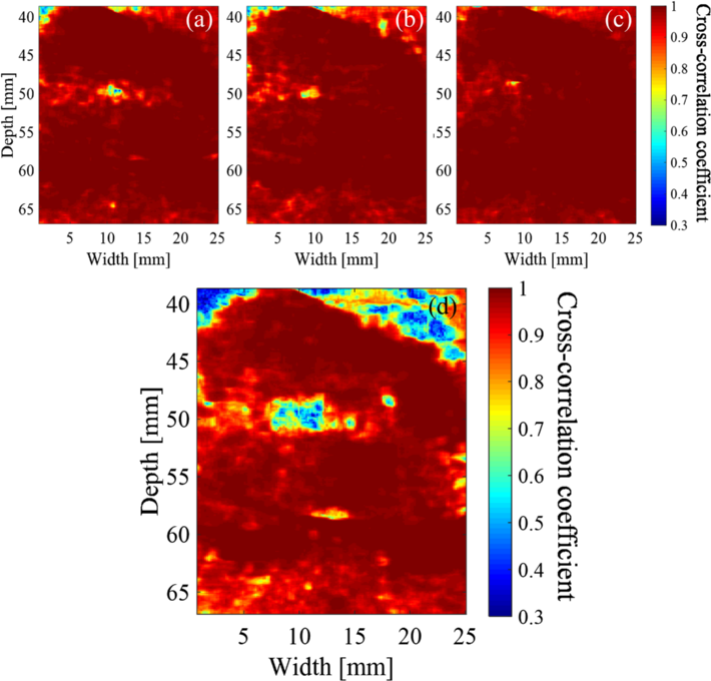
Distributions of cross-correlation coefficient with 50-ms intervals. **a** 2.5 s, **b** 5.0 s, and **c** 9.0 s after start of HIFU exposure and **d** the minimum hold values from 0.5 to 10 s

**Fig. 8:**
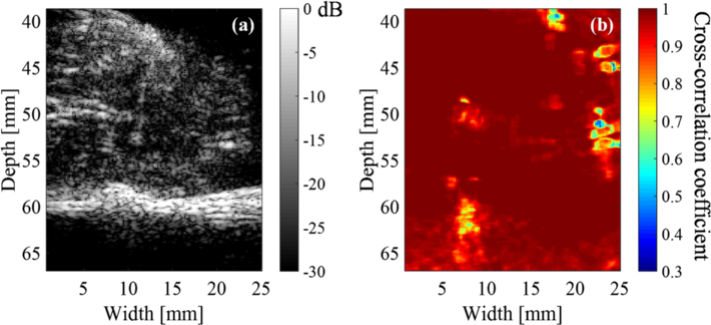
Results after HIFU exposure without trigger pulses. **a** B-mode image of the tissue at the end of the HIFU exposure and **b** distribution of minimum hold cross-correlation coefficients with 50-ms intervals

The distributions of the maximum cross-correlation coefficient with 50-ms intervals are shown in Fig. 7. The tissue sample is the same that was used in Figs. 5 and 6. Slightly decreased correlation is seen at (a) 2.5 s, (b) 5.0 s, and (c) 9.0 s after the start of the HIFU exposure sequence. The minimum values of the cross-correlation coefficients from 0.5 to 10 s were mapped in Fig. 7d. The values of cross-correlation coefficients before 0.5 s were not included in the minimum hold map because low cross-correlation coefficients were observed in a large region, probably caused by the tissue motion and deformation due to the acoustic radiation force of HIFU. The areas of low correlation (less than about 45 mm in depth) in Fig. 7d correspond to the water between the probe and the tissue. Figure 8 shows the B-mode image after the HIFU exposure and the distribution of the minimum hold cross-correlation coefficients with 50-ms intervals, for another sample exposed without trigger pulses. Cavitation bubbles were not observed in ultrasound images in this case. Figure 8b, in contrast with Fig. 7d, demonstrates that almost all coefficients around the focal spot of HIFU, 50.0 mm in depth, were larger than 0.9.

### Distribution of cross-correlation coefficient with 500-ms intervals

**Fig. 9:**
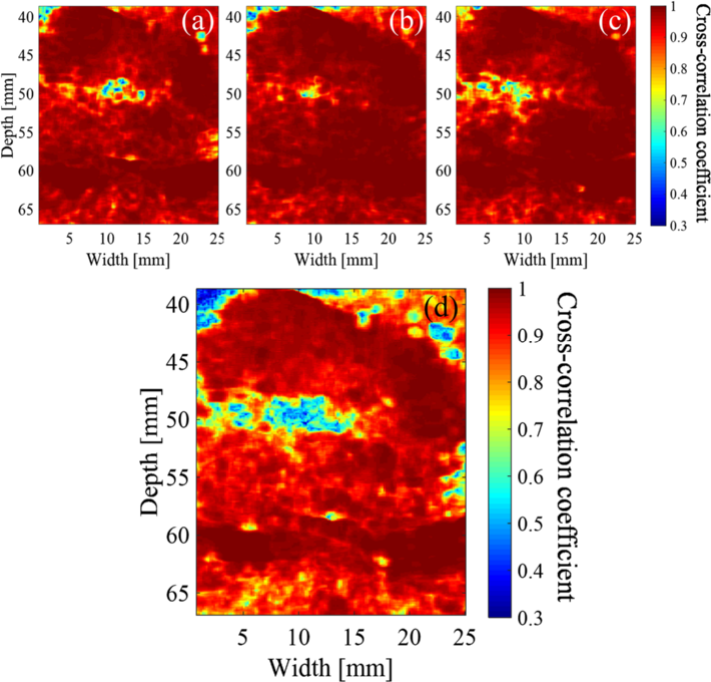
Distributions of cross-correlation coefficient with 500-ms intervals, **a** 2.5 s, **b** 5.0 s, and **c** 9.0 s after start of HIFU exposure and **d** minimum hold distribution from 1 to 10 s

Figure 9 shows the distributions of cross-correlation coefficient with 500-ms intervals. The tissue sample is the same that was used in Figs. 5, 6, and 7. A decrease in correlation is seen at (a) 2.5 s, (b) 5.0 s, and (c) 9.0 s after the start of the exposure sequence. The minimum values of the maximum cross-correlation coefficients with 500-ms intervals from 1 to 10 s are mapped in Fig. 9d. The values of cross-correlation coefficients at 0.5 s were not included in the minimum hold map.

### Cross-correlation coefficient threshold

**Fig. 10:**
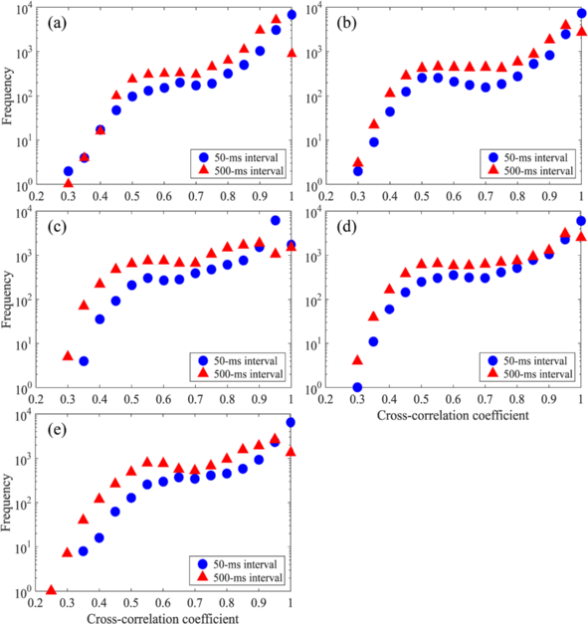
Histograms against cross-correlation coefficients of **a** sample (1), **b** sample (2), **c** sample (3), **d** sample (4), and **e** sample (5) with 50-ms intervals (*blue circles*) and 500-ms intervals (*red triangles*)

Figure 10a–e shows the histograms of five samples (1)–(5), respectively. The tissue sample used in Figs. 5, 6, 7, and 9 corresponds to sample (1). The range of depth and width to count pixels was set −6.0 to 6.0 mm and −9.0 to 6.0 mm from the HIFU focal point, respectively, resulting in a frame size of 142 by 90 pixels. The plots of blue circles and red triangles show the histograms of cross-correlation coefficients with 50- and 500-ms intervals, respectively. The cross-correlation coefficient thresholds for 50-ms intervals determined from the corresponding histogram in Fig. 10a–e were 0.7, 0.7, 0.6, 0.7, and 0.7, respectively. That with 500-ms intervals was 0.7, 0.75, 0.7, 0.65, and 0.7, respectively.

### Size of decorrelated area

**Fig. 11:**
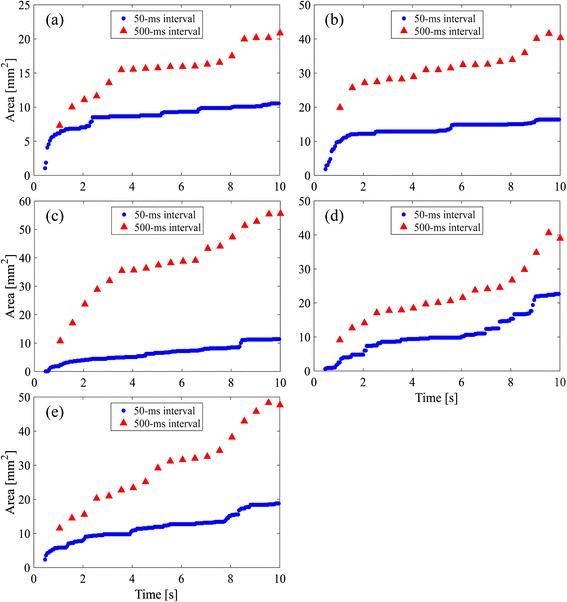
Estimated areas of cavitation and coagulation of **a** sample (1), **b** sample (2), **c** sample (3), **d** sample (4), and **e** sample (5) with 50-ms intervals (*blue circles*) and 500-ms intervals (*red triangles*)

Figure 11a–e shows the areas of decorrelated region plotted against HIFU exposure duration for each one of the five samples. The plots of blue circles and red triangles show areas where the minimum hold cross-correlation coefficients with 50- and 500-ms intervals, respectively, are lower than the thresholds determined from Fig. 10. The coagulation area estimated in Fig. 11a–e at HIFU exposure duration of 10 s with 500-ms intervals was about 21, 40, 55, 40, and 48 mm^2^, respectively.

### Comparison between distribution of cross-correlation coefficient with 50-ms intervals and pulse inversion image

**Fig. 12:**
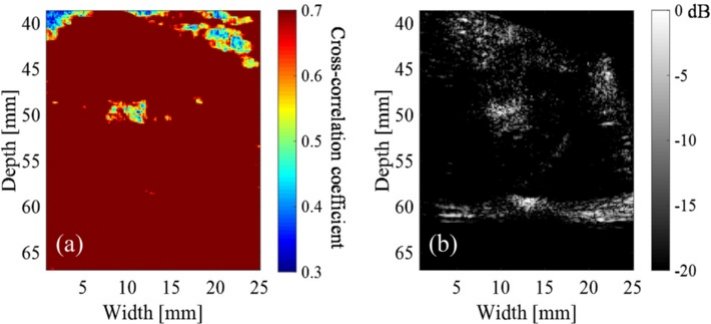
**a** Distribution of minimum hold cross-correlation coefficients of sample (1) with 50-ms intervals, when the cross-correlation coefficient threshold is set at 0.7, and **b** PI image of the sample at the end of the HIFU exposure

Figure 12a shows the distribution of minimum hold cross-correlation coefficient of sample (1) with 50-ms intervals. The upper range of the color bar, 0.7, was the threshold determined from Fig 8a. It is compared with the corresponding PI image shown in Fig. 12b, depicting cavitation bubbles at the same location with decreased coefficients as in Fig. 12a.

### Comparison between distribution of cross-correlation coefficient with 500-ms intervals and histology

Table 1 shows the coagulation area, *A*_HE_, where the coarse region of atrophic muscle fibers was observed in histology, the coagulation area, *A*_US_, estimated from the cross-correlation coefficients with 500-ms intervals for the threshold determined from the histogram, and the ratio between them, *A*_US_/*A*_HE_, for the 5 samples, (1)–(5). The ratio is within 100 ± 25 % for all the samples.

**Table 1:** The comparison between the observed and estimated coagulation area

	(1)	(2)	(3)	(4)	(5)
Observed coagulation area in histology, *A*_HE_ [mm^2^]	26	32	48	48	59
Estimated coagulation area, *A*_US_ [mm^2^]	21	40	55	40	48
Cross-correlation coefficient threshold for 500-ms intervals	0.7	0.75	0.7	0.65	0.7
Ratio of *A*_US_ to *A*_HE_ (%)	81	125	114	83	81

### Comparison between distributions of cross-correlation coefficient with 50- and 500-ms intervals

**Fig. 13:**
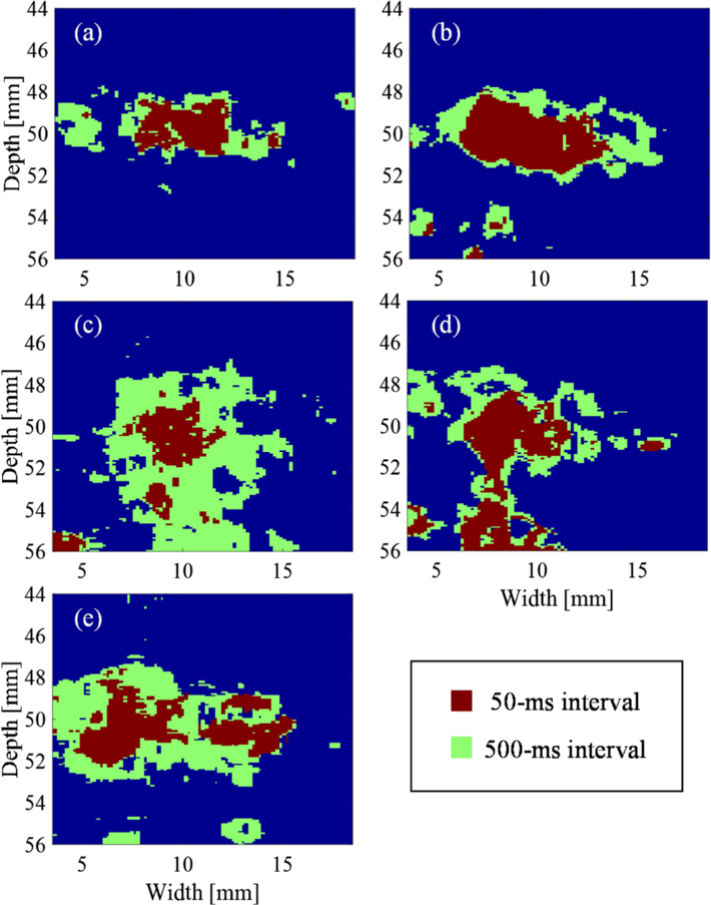
Areas of minimum hold cross-correlation coefficients with 50- and 500-ms intervals less than thresholds determined from histograms of **a** sample (1), **b** sample (2), **c** sample (3), **d** sample (4), and **e** sample (5)

Figure 13a–e shows the decorrelated areas for the samples (1)–(5), respectively. The areas plotted in red and green are those with the minimum hold cross-correlation coefficient with 50- and 500-ms intervals, respectively, lower than the threshold determined from the corresponding histogram. The red areas with 50-ms intervals are mostly contained by those with 500-ms intervals.

## Discussion

### Cavitation region

Figure 8b demonstrates that the correlation coefficient particularly decreased with 50-ms intervals at the focal spot of HIFU with a sequence inducing cavitation while it hardly decreased with a sequence inducing no cavitation. Furthermore, Fig. 12 shows that the distribution of minimum hold cross-correlation coefficients agrees with the corresponding PI image. As the trigger pulses were exposed every 50 ms, RF echo from cavitation bubbles could change significantly in 50 ms, which should cause the decrease of the cross-correlation coefficients. Therefore, it is necessary to detect the decorrelation area with 500-ms intervals outside the area with 50-ms intervals as shown in Fig. 12a for the coagulation detection when RF echo from cavitation bubbles cannot be neglected.

### Coagulation region

The decrease in correlation coefficient with 500-ms intervals has a potential to be used for estimating the region of tissue where coagulation occurs even when cavitation is employed, because of the following reasons. First, the size of the estimated areas agreed well with the coagulation areas observed in histology as shown in Table 1. Second, all the areas regarded as those of cavitation are contained in the estimated coagulation areas as shown in Fig. 13. Therefore, the proposed way of estimating a coagulation area does not seem to significantly suffer from the decrease in correlation due to cavitation, at least, in the exposure sequences as tested.

The coagulation area from histology of sample (1) in Table 1 contains an area where the muscle fibers are a little denser than other coagulated areas, which can be seen in lower right area in Fig. 6a. The temperature rise in the denser area might be lower in other coagulated areas. The denser area approximately corresponds to that around a depth and width of 50 and 6 mm, respectively, in Fig. 13a, where the correlation coefficients are larger than the thresholds for 50- and 500-ms interval data. As a result, the denser area in Fig. 6a was included in *A*_HE_ but not in *A*_US_ as shown in Fig. 13a. If such an area in *A*_HE_ is eliminated from the calculation, the ratio, *A*_US_/*A*_HE_, will become to 98 %, much closer to 100 %. The decorrelation detection using histograms in this study is based on the detection of the significant change in tissue structure due to coagulation. If there are no significant changes in tissue structure, it should be difficult to detect. Therefore, the method may underestimate the treated region. It may be suitable for monitoring to ensure the therapeutic effects of HIFU thermal treatments but may not be sufficient for monitoring the side effects. Also, the method may be applicable to the monitoring of lesion formation in histotripsy, where tissues are mechanically fractionated and the tissue changes probably cause the decorrelation of the ultrasound echo signals.

Figure 13b shows a decorrelated area ranging in a depth of 54–56 mm, and a width of 3.5–10-mm point is not in the HIFU focal zone. This decorrelated region is thought to have been due to the motion of preexisted bubbles. Figure 13c, d also shows decorrelated areas below the HIFU focal zone. These decorrelated regions are thought to have been caused by a kind of acoustic shadows of the cavitation bubbles in the HIFU focal zone.

The coagulation volume in a depth of 47–53 mm and a width of 4.5–18 mm in Fig. 13b was estimated to be 85 mm^3^ using the following formula as

3 $$ \left(\mathrm{volume}\right)=\frac{\pi }{6}\times \left(\mathrm{major}\;\mathrm{axis}\right)\times {\left(\mathrm{minor}\;\mathrm{axis}\right)}^2 $$

Here, the shape of the coagulation volume is assumed to be a spheroid and the axis of symmetry is parallel to the propagation of HIFU. The coagulation volume estimated from the H&E-stained tissue sample was 92 mm^3^. Although the estimated coagulation volume from the decorrelation shows a good agreement with that estimated from H&E-stained tissue sample in the case of Fig. 13b, the reasonable volume estimation seems to be difficult especially in the case of Fig. 13c, d because of the complicated shape of the decorrelated areas.

### Temporal change in estimated areas

The temporal change in the estimated areas shows a tendency common for the 5 samples in Fig. 11a–e. The change has the following five features I–IV as shown in Fig. 11.

I. The estimated area with 50-ms intervals increased quickly for the first 3 s of the exposure and gradually after that. This is interpreted that the cavitation bubbles were generated only near the HIFU focal point for the first 3 s, and then they started being generated in the area migrating toward the HIFU transducer [27].

II. The estimated area with 500-ms intervals increased even more quickly than that with 50-ms intervals from 1 to 4 s after the start of the exposure. This is interpreted that tissue coagulation started and expanded near the focal point in this time zone.

III. The estimated area with 500-ms intervals increased gradually at a similar slope as with 50-ms intervals from 4 to 8 s after the start of the exposure. This is interpreted that they increased similarly only due to cavitation for a while after the rapid coagulation near the focal point has stopped.

IV. The estimated area with 500-ms intervals increased quickly from 8 s after the start of the exposure. This is interpreted that coagulation started a little distance away from the focal point. For example, the decorrelation area around a depth and width of 50 and 5 mm in Fig. 13a was observed in this time zone, as shown in Fig. 9c, while the decrease in correlation coefficients to below the threshold with 50-ms intervals are not particularly observed in the area as shown in Fig. 7c. This result suggests that a part of the decorrelation areas with 500-ms intervals outside those with 50-ms intervals could be detected without the influence of the decorrelation due to cavitation bubbles, as seen in this time zone, resulting in a good agreement between the observed and estimated coagulation area.

## Conclusions

In this study, ultrasonic RF signals during HIFU exposure were used to estimate the cavitation and coagulation regions by calculating cross-correlation coefficients between IQ data of RF frames with certain intervals. The results show that the coagulation region was approximately estimated from the decorrelation map with 500-ms intervals and the size of decorrelated area in the map matched that of the actual coagulation area observed in histology with a cross-correlation coefficient threshold set properly. The estimation of coagulation regions with 500-ms intervals outside decorrelated regions with 50-ms intervals was not significantly affected by cavitation preceding the coagulation, at least in a certain HIFU sequence. This method for the coagulation detection will contribute to improve the safety and accuracy of the HIFU treatment enhanced by cavitation microbubbles.
